# Legal challenges to the 340B drug pricing program: administration, regulation, and reform

**DOI:** 10.1093/haschl/qxaf125

**Published:** 2025-06-25

**Authors:** Ryan P Knox, Ameet Sarpatwari

**Affiliations:** College of Law, DePaul University, 25 E. Jackson Blvd, Chicago, IL 60604, United States; Division of Health Policy and Insurance Research, Department of Population Medicine, Harvard Pilgrim Health Care Institute and Harvard Medical School, Boston, MA 02215, United States

**Keywords:** 340B Drug Pricing Program, prescription drugs

## Abstract

The 340B Drug Pricing Program (“340B Program”) was created to help safety net hospitals and clinics “stretch scarce federal resources” by discounting the price of their outpatient drug purchases. The spread between the discounted price and the sales price of the drug is retained by the hospitals and clinics, subsidizing operational costs and funding services that would not otherwise be possible to provide. The 340B Program has grown dramatically since its inception, eliciting significant criticism and calls for reform. Many legal challenges against the 340B Program and the agency tasked with administering the program—the Health Resources and Services Administration (HRSA)—have been brought, highlighting the limitations in HRSA's powers. Specifically, HRSA's lack of broad regulatory authority has hampered its ability to oversee and reform the 340B Program. This article analyzes the litigation challenging the 340B Program—including cases regarding the orphan drug exclusion, the definition of a 340B patient, and the use of contract pharmacies—and explores the scope of HRSA's authority to regulate the 340B Program. In doing so, this article emphasizes the need for reforms to ensure that the 340B Program operates consistent with its congressional purpose and patients maintain access to safety net care across the country.

## Introduction and background

The 340B Drug Pricing Program (“340B Program”) was created to help safety net hospitals and clinics “stretch scarce federal resources” by discounting the price of their outpatient drug purchases, thereby subsidizing their provision of healthcare services.^1^ It was first implemented as a response to the 1990 implementation of the Medicaid Drug Rebate Program, which requires manufacturers participating in Medicaid to pay rebates on purchases of drugs by state Medicaid programs.^1,2^ Medicaid rebates are set at the lower of a statutorily defined percent discount or the best price which the manufacturer has sold the drug to a non-federal payer.^3^ Prior to the Medicaid Drug Rebate Program, manufacturers commonly sold drugs to safety net hospitals, veterans’ hospitals, and federally funded clinics at substantial discounts.^4,5^ However, due to concerns over the best price rule, manufacturers stopped these discounts after the enactment of the Medicaid Drug Rebate Program.^1,3^ Safety net hospitals and clinics experienced significant price increases on drug purchases, ranging from 21% to 32%, which strained their budgets and threatened their ability to continue providing care to patients at previous levels.^1^

The 340B Program responded to the needs of safety net hospitals and clinics by statutorily providing discounts on their purchases of outpatient drugs. The 340B discounted price is linked to the Medicaid price for the drug,^6,7^ with most discounts ranging from 20% to 50% of the average manufacturer price.^8^ The spread between the discounted price and the sales price of the drug is retained by the hospitals and clinics, subsidizing operational costs and funding services that would not otherwise be possible to provide.^9^ Safety net clinics have reporting requirements associated with their status as a federal grantee, but most hospitals do not.^8,9^ Further, there are no set requirements on how the revenue generated from the 340B Program is used by participating hospitals and clinics.^8,9^

The 340B Program has grown dramatically since its inception. In 1992, there were approximately 1000 hospitals and clinics, called “covered entities,” participating.^10^ In 2021, this increased to 50 000 covered entities.^10-12^ Much of the recent growth of the 340B Program can be attributed to pharmacies dispensing drugs on behalf of covered entities, called “contract pharmacies.” The number of contract pharmacies participating in the 340B Program increased from <1300 in 2010 to more than 33 000 in 2023.^10-13^

The rapid growth of the 340B Program has received significant criticism. Proponents of the 340B Program argue that covered entities would be unable to run without 340B revenue and that the program provides valuable benefits to patients in the form of increased access to healthcare services.^9,14^ Critics, however, challenge the oversight and integrity of the 340B Program and argue the program's expansion has been inconsistent with the program's intended purpose: benefitting underserved patients.^15,16^

Many legal challenges against the 340B Program and the agency within the Department of Health and Human Services (HHS) tasked with administering the program—the Health Resources Services Administration (HRSA)—have been brought, highlighting the limitations in HRSA's regulatory authority.^17^ These limitations have hampered HRSA's ability to effectively oversee and reform the 340B Program. Reform to the agency's authority is needed to permit HRSA to take an active role in ensuring that the 340B Program operates consistently with its congressional purpose and that patients maintain access to safety net care across the country.

This article analyzes the litigation challenging the 340B Program and examines the scope of HRSA's authority to regulate the 340B Program. It finds that courts have held that HRSA has limited authority to make binding rules regarding the administration of the 340B Program. In doing so, this article emphasizes the need for legislative reforms at the federal and state levels to increase the oversight and improve the integrity of the 340B Program.

## Litigation against the 340B drug pricing program

Several cases have been brought challenging HRSA's authority to regulate and enforce obligations under the 340B Program. These cases have involved three aspects of the 340B Program—the orphan drug exclusion, the definition of a 340B patient, and the use of contract pharmacies. Each of these cases identifies a gap in HRSA's authority to issue binding regulations, leading to disagreements regarding 340B compliance and obligations. Collectively, these cases demonstrate HRSA's lack of broad authority to regulate and administer the 340B Program and the associated consequences.

### The orphan drug exclusion litigation

The limitations in HRSA's regulatory authority were first seen in a challenge to the agency's interpretation of drugs included in the 340B Program's mandatory discounts. In 2010, the Patient Protection and Affordable Care Act added four new hospital categories of covered entities.^18^ Unlike other 340B covered entities, hospitals in these four categories were ineligible to purchase “orphan drugs,” or drugs “designated by… [the FDA for treatment of] a rare disease or condition,” at 340B discounts.^6^ This was significant as orphan drugs can cost thousands of dollars a month and include some notable high-cost blockbuster drugs, including adalimumab (Humira).^17^ Excluding orphan drugs from parts of the 340B Program, therefore, is financially beneficial to the pharmaceutical industry and decreases potential 340B revenue for hospitals and clinics.

Recognizing this limitation to the orphan drug exclusion, HRSA argued in a notice of proposed rulemaking that orphan drugs were only excluded for the new covered entities when the “orphan drugs that [we]re transferred, prescribed, sold, or otherwise used for the rare condition or disease” as opposed to other potential indications.^20^ This interpretation made some orphan drugs eligible for 340B discounts, to the benefit of these covered entities and to the detriment of the pharmaceutical industry.

Pharmaceutical Research Manufacturers of America (PhRMA), the major lobbying group for the pharmaceutical industry, sued HRSA in 2014, claiming the agency lacked authority to issue the orphan drug exclusion rule. The court agreed with PhRMA and defined HRSA's regulatory authority narrowly as limited to “(1) the establishment of an administrative dispute resolution (ADR) process, (2) the “regulatory issuance” of precisely defined standards of methodology for calculation of ceiling prices, and (3) the imposition of monetary civil sanctions” for manufacturer overcharging of covered entities.^19^ The orphan drug exclusion did not fall under these three categories and was invalidated by the court. Perhaps more importantly, the court's opinion influenced future challenges to HRSA's authority, carrying on a narrow view of when the agency can issue binding regulations and a less deferential attitude towards HRSA's regulatory actions.

### Litigation on the definition of 340B patients

The limitations of HRSA's regulatory authority arose again regarding a challenge to the definition of a “patient” of a 340B covered entity.^21,22^ Covered entities are permitted to dispense 340B discounted drugs to their patients, but are prohibited from diverting these drugs to other patients and buyers.^6^ Non-compliance due to diversion is widespread, with 44% of audits of covered entities conducted between 2012 and 2019 reporting diversion violations.^23^ This non-compliance finding indicates thousands of ineligible prescriptions purchased at 340B discounts.

Although diversion refers to dispensing drugs to ineligible patients, the term “patients” is not defined by the 340B statute, complicating compliance with the diversion prohibition. To clarify this ambiguity, HRSA issued guidance in 1996 stating that individuals qualify as 340B patients if “(1) the covered entity maintains records of the individual's care; (2) the individual receives care from a healthcare professional employed by or contracted with the covered entity; and (3) for grantee clinic covered entities, that the care the individual receives is consistent with the services making the entity eligible for federal funding.”^24^

This definition of a 340B patient was challenged in 2019 by Genesis Healthcare, a covered entity that was removed from the 340B Program for alleged compliance violations related to dispensing drugs to ineligible patients.^25^ Genesis argued that HRSA's additional requirements to qualify as a patient set forth in the 1996 guidance were inconsistent with the 340B statute. The court sided with Genesis.^25^ Because HRSA did not have authority to make binding rules related to the definition of a patient, the court applied a less deferential standard of review to HRSA's interpretation. The court ultimately held that the “only statutory requirement for 340B eligibility of a person is that the person be a patient of a covered entity.”^25^ No additional clarification or definition was given by the court however, leaving continued ambiguity in who qualifies as a patient of a 340B covered entity and HRSA unable to fill this gap.

### Contract pharmacies litigation

Additional challenges to the 340B Program have been related to covered entities’ use of contract pharmacies to dispense drugs to patients on the covered entities’ behalf.^26-29^ Although the 340B statute does not speak to the use of pharmacies, contract pharmacies have been involved in the 340B Program since its inception, and HRSA has issued multiple guidance documents permitting their participation.^17^ HRSA began by allowing one contract pharmacy per covered entity in 1996 and increased this to an unlimited number of contract pharmacies in 2010.^30,31^ In 2019, there were almost 25 000 contract pharmacies generating $5 billion in profit annually; as of 2023, there were over 30 000 contract pharmacies.^13,32^

In 2020, several pharmaceutical manufacturers announced that they would no longer comply with HRSA's directive to deliver drugs to an unlimited number of contract pharmacies.^33-35^ For most covered entities—particularly hospital covered entities—manufacturers set restrictions on their participation in the 340B Program. Some companies restricted delivery of 340B discounted drugs to a single contract pharmacy; others required covered entities submit claims data for contract pharmacy prescriptions; and others restricted only certain drugs from the 340B Program.^36-38^ Manufacturers argued that the use of contract pharmacies increased the risk of diversion as well as duplicate discounting—selling a drug at a 340B discount and also paying a Medicaid rebate on the drug—both of which are prohibited in the 340B Program.^17^ The risks of diversion and duplicate discounting are supported by HRSA, which found diversion in 44% and duplicate discounting in 35% of audits between 2012 and 2019.^23^

In response to manufacturers’ restrictions, HHS Office of the General Counsel issued an Advisory Opinion and HRSA sent individual Violation Letters to each manufacturer, both asserting that manufacturers were required to deliver 340B discounted drugs purchased by covered entities to an unlimited number of their contract pharmacies.^39-46^ Six manufacturers—AstraZeneca, Eli Lilly, Novartis, Novo Nordisk, Sanofi, and United Therapeutics^26-29^—then brought actions against HRSA, challenging both the Advisory Opinion and the Violation Letters, asserting that HRSA lacked the authority to issue these requirements. Courts reviewing challenges have all ultimately agreed with manufacturers that some conditions or restrictions are permitted on their participation in the 340B Program and therefore vacated the Advisory Opinion and the Violation Letters.^12,26-29,47^ The courts recognized the ambiguity in the 340B statute and HRSA's lack of authority to issue rules prohibiting such conditions on participation in the 340B Program. The U.S. Court of Appeals for the D.C. Circuit, for example, emphasized that “[t]he Secretary lacks rulemaking authority over the section 340B [P]rogram” and held that although some restrictions could violate the 340B statute, all conditions were not “categorically prohibit[ed].”^47^ As a result, HRSA was unable to require manufacturer compliance with its contract pharmacy guidance and the manufacturer restrictions remained in place, limiting access to 340B discounted drugs.

## Implications of limitations in HRSA's regulatory authority

The court's decisions in these cases show that HRSA's regulatory authority extends only to the three areas identified in the orphan drug exclusion litigation: the ADR process, ceiling price calculations, and civil monetary penalties for manufacturer overcharges.^17^ Outside of these issues, HRSA lacks authority to impose binding regulations clarifying the requirements of the 340B Program. This narrow authority is particularly concerning given the ambiguities in the 340B statute. Major details about how the 340B Program should operate and be administered—from how discounted drugs are offered, delivered, and tracked to which patients are eligible to receive discounted drugs—are absent from the 340B statute. As a result, covered entities and pharmaceutical companies alike are left guessing and unsure if they are in compliance with program requirements, particularly with the 340B Program's duplicate discounting and diversion prohibitions. And as seen in these cases, HRSA cannot issue binding rules to clarify these ambiguities and resolve most of the problems stemming from the statutory ambiguities.

The outcomes of these cases, and in turn HRSA's limited regulatory authority, have real consequences for the 340B Program and its stakeholders. In the Genesis case, the definition of a patient adopted by the court allows for a much broader pool of patients to be eligible for 340B discounted drugs. One study estimated this may increase 340B profits for covered entities by 33% to $16 billion for Medicare Part D prescriptions alone.^49^ Another study found a particularly expansive interpretation of the definition of patient could increase 340B profits on Medicare Part D prescriptions by up to $55 billion, with contract pharmacies contributing to much of that growth.^50^ In the case of the contract pharmacies litigation, the courts’ decisions results in fewer pharmacies permitted to participate in the 340B Program. The five largest contract pharmacy companies estimated that manufacturer restrictions would decrease their gross profits from $3.2 billion in 2021 to $2.9 billion in 2023.^13^ These cases may also have broader implications on the pharmacy market more generally, including affecting incentives for vertical integration between pharmacies and health plans, pharmacy benefit managers, and pharmacy administrators.^48^

Litigation challenging the 340B Program is ongoing, and stakeholders continue to take action to modify the scope of the program. The definition of a patient is still yet to be conclusively determined, and manufacturers have continued to impose restrictions on their participation in the 340B Program related to contract pharmacies. Furthermore, some pharmaceutical companies have chosen to rework their participation in the 340B Program from one of upfront discounts, as the program was designed, to a rebate model, putting additional compliance obligations on the covered entities intended to benefit from the program.^38^ Without reforms at the federal and state levels, ambiguities regarding the requirements of the 340B Program will continue and disputes over these issues will be left to interpretation independently by the courts instead of expert agencies’ regulatory decision-making.

## Recommendations for reform

Reforms at the federal and state levels are likely needed to ensure the 340B Program operates consistently with Congress’ intent to support safety net hospitals and clinics in providing continued care to patients in need across the country.

Some reforms have already been introduced or implemented to address some of the ongoing problems in the 340B Program. Some have been accomplished by federal agency rulemaking. For example, in 2018, the Centers for Medicare and Medicaid Services added a 340B claims modifier to Medicare Part B billing to help prevent diversion and duplicate discounts.^51^ In 2024, HRSA reformed the ADR process for the 340B Program, enhancing the ability of covered entities and manufacturers to resolve claims more efficiently and transparently.^52^ While these actions have been effective in addressing some specific problems posed by the 340B Program, given HRSA's lack of rulemaking authority over most of the ambiguities present in the 340B Program, most needed reforms cannot be accomplished by agencies alone.

In light of this, some reforms have been implemented at the state level. At least eight states, for example, have passed laws to protect the use of contract pharmacies in the 340B Program in their states.^53^ Other states have passed laws protecting covered entities from receiving lower reimbursements for 340B discounted drugs from payers and pharmacy benefit managers.^54^ States have also implemented a range of laws and policies governing the intersection between the 340B Program and state Medicaid programs to prevent duplicate discounting and diversion.^55,56^ Particularly in the absence of federal reform, and while HRSA's authority to resolve the ambiguities in the 340B Program remains limited, states should continue to implement laws and policies filling the current gaps in HRSA's regulatory authority.

Ultimately, however, 340B Program reform must come from Congress. A range of specific legislative fixes and broad regulatory changes should be implemented in order to improve operation and administration of the 340B Program and to strengthen HRSA's ability to oversee and enforce 340B Program requirements.

First, Congress should add provisions to the 340B statute defining both patients of 340B covered entities and articulating the role of contract pharmacies in the 340B Program.^19^ Congress should clarify how close a relationship must exist between a covered entity and an individual receiving a 340B discounted drug and why additional guardrails are needed here. Congress should also define contract pharmacies as agents of covered entities, consistent with their current participation in the 340B Program. Doing so would help resolve key ongoing disputes in the 340B Program by clarifying patient eligibility and the permissibility of contract pharmacy arrangements.

Second, Congress should delegate expanded regulatory authority to HRSA so that it can adequately regulate and oversee the 340B Program.^17^ While Congress clarifying patients’ and contract pharmacies’ roles in the 340B Program provides a short term solution, other disputes are likely to arise. Given the complexities of the 340B Program, HRSA is best suited to resolve ongoing controversies, yet lacks the necessary authority to do so. Broader rulemaking and enforcement authority would allow HRSA to issue binding rules, addressing issues as they arise or proactively resolving identified or anticipated ambiguities. For example, HRSA could issue rules setting policies to prevent duplicate discounting and diversion, including recordkeeping requirements, and requirements on reporting how covered entities spend their 340B revenue. Such actions would help improve administration and oversight of the 340B Program in the long term.^9^

Without reforms at the federal and state levels, Congress’ intent for the 340B Program—to promote and protect access to care for patients who rely on safety net providers—could be jeopardized. Reforms are needed to strengthen HRSA's regulatory authority so that it may effectively oversee, administer, and enforce the requirements of the 340B Program, allowing it to operate as Congress intended.

## Conclusion

The 340B Program provides drug discounts to safety net providers serving low-income and underserved populations. Limitations in HRSA's regulatory authority have impaired its ability to oversee and administer the 340B Program. Reforms at the federal and state levels clarifying existing ambiguities in the program and expanding HRSA's regulatory authority are necessary to ensure that the 340B Program operates consistent with Congress’ intent and can maintain access to care for patients across the country.

## Supplementary Material

qxaf125_Supplementary_Data
